# Substituted Benzamides Containing Azaspiro Rings as Upregulators of Apolipoprotein A-I Transcription

**DOI:** 10.3390/molecules17067379

**Published:** 2012-06-14

**Authors:** Yu Du, Yuan Yang, Wei Jiang, Li Wang, Xiao-Jian Jia, Shu-Yi Si, Xiao-Fang Chen, Bin Hong

**Affiliations:** Institute of Medicinal Biotechnology, Chinese Academy of Medical Sciences & Peking Union Medical College, Tian Tan Xi Li 1#, Beijing 100050, China

**Keywords:** apolipoprotein A-I, transcriptional upregulator, structure-activity relationship, atherosclerosis

## Abstract

Apolipoprotein A-I (Apo A-I) is the principal protein component of high density lipoprotein (HDL), which is generally considered as a potential therapeutic target against atherosclerosis. The understanding of the Apo A-I regulation mechanism has fuelled the development of novel HDL targeted therapeutic approaches. To identify novel agents that can upregulate Apo A-I expression, we performed a cell-based reporter assay to screen 25,600 small molecules. Based on the dataset obtained from screening, a series of novel analogs of substituted benzamides containing azaspiro rings were assessed for their ability to induce the transcription of the Apo A-I gene, and the structure-activity relationship (SAR) around these analogs was also proposed. The results indicated that the trifluoromethyl substituted benzamide containing an azaspiro ring is a promising backbone for designing Apo A-I transcriptional upregulator and could be viable leads for development of new drugs to prevent and treat atherosclerosis in the future.

## 1. Introduction

Numerous epidemiologic studies have demonstrated an inverse relationship between plasma high density lipoprotein (HDL) levels and the risk of atherosclerotic cardiovascular diseases (CVD) [1,2,3], and targeting HDL therapeutically presents an attractive strategy towards treating CVD. Apolipoprotein A-I (Apo A-I) is the major and critical protein component of HDL particles and responsible for reverse cholesterol transport (RCT) [4,5], transporting cholesterol from peripheral tissues to the liver for further metabolism and excretion in the form of bile salts or free cholesterol [6]. Thus enhancing the endogenous expression of Apo A-I is expected to reduce the risk of atherosclerosis. Overexpression of human Apo A-I in transgenic mice has elucidated that increased Apo A-I and HDL-cholesterol (HDL-C) reduced atherosclerosis progression and presented other anti-atherogenic effects [7,8]. On the basis of these observations, the development of new therapies targeted to Apo A-I/HDL has been greatly promoted. For example, admin-istration of recombinant HDL (lipid-poor Apo A-I/phospholipid complexes) or purified native Apo A-I/phospholipids increased Apo A-I and HDL-C levels, cholesteryl ester transfer protein (CETP) inhibitor indirectly raised Apo A-I levels, and so on [9]. Furthermore, small molecules that could upregulate Apo A-I gene expression may be a promising approach to target HDL, and such a compound termed RVX-208 has being evaluated in early-phase clinical trials [10].

To identify novel small molecular upregulators of Apo A-I transcription, we performed a cell-based reporter assay [11] against more than 25,600 small molecules. Several substituted benzamides containing azaspiro rings were identified as hit compounds. Then a series of analogs of the compounds were selected from the screening library and retested to evaluate the structure-activity relationship (SAR). The results arising from this work indicated the potential structure features of upregulating Apo A-I transcription. These findings will provide clues to identify more potent analogs and provide access to more drug-like compounds to raise transcriptional levels of Apo A-I.

## 2. Results and Discussion

### 2.1. Screening for Compounds Upregulating Apo A-I transcription

Though several known drugs and other compounds, such as fenofibrate, pioglitazone, and 9-*cis* retinoic acid, have been reported to regulate Apo A-I gene transcription by activating peroxisome proliferators-activated receptor α (PPARα) or retinoid X receptor (RXR) and retinoic acid receptor (RAR) heterodimer [12,13,14], the upregulation capacity of these compounds has not been as potent as expected, so identification of effective small molecules that raise the Apo A-I production by increasing its transcription is highly desired. In a previous study, we developed a cell-based assay to identify novel small molecule compounds with a stable HepG2 strain that expresses the reporter gene driven by human Apo A-I gene promoter [11]. Resveratrol, a plant polyphenol, was used as the positive control. The dose response curve for resveratrol exhibits a 3.69 fold maximum upregulation fold at 5 μg/mL. Resveratrol has been reported to increase Apo A-I gene transcription and the promoter activity in HepG2 cells [15]. Based on its structure, RVX-208 was developed and provided some encouraging results in the ongoing clinical trial [16]. The assay performance measures, such as signal-to-background ratio (S/B), signal-to-noise ratio (S/N) and Z'-factor were assessed. The values of S/B, S/N and Z'-factor were 5.12, 30.1 and 0.68, indicating the assay satisfied the demands of high throughput screening. Using this cell-based reporter assay, over 25,600 compounds were screened for their ability to induce Apo A-I transcription in the present study. Following the screening procedure (described in the Experimental), compounds with consistent upregulatory activities higher than 1.5-fold were selected for further consideration. At this stage, 255 small molecule compounds were found positively responded from the screening and were thus considered primary hits.

### 2.2. Biological Activity of Substituted Benzamide Containing Aza Spiro Ring Analogs

In our study, several novel compounds identified from screening as possessing significant activity share some common pharmacophore characters, which can be divided into two parts: (i) a substituted benzoyl moiety, (ii) an azaspiro ring. Then all the compounds with the similar skeleton structure were selected from the initial screening library and re-evaluated at the concentration of 5 μg/mL and 10 μg/mL, respectively. The results were shown in the Table 1 and all of these compounds showed no toxicity in HepG2 cells at the indicated concentrations. The most potent compounds were A-2, B-1 and E-2 with about 3-fold induction activity at 5 μg/mL and 10 μg/mL. So far, five types of spiro rings were found to have potential to show good activity, such as 2,8-diazaspiro[4.5]dec-2-yl (A), 2,7-diaza-spiro[4.4]non-2-yl (B), 2,8-diaza-spiro[4.5]dec-8-yl (C), 1-oxa-4,8-diaza-spiro[5.5]undec-8-yl (D), and 1-oxa-4,8-diaza-spiro[5.5]undec-4-yl (E).

**Table 1:** Induction of Apo A-I transcription in HepG2 cells. 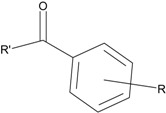

Compound entry		R'	R	Activity (fold induction)	
In series	In library			5 μg/mL	10 μg/mL
A-1	4011061	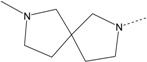	3-CF_3_	1.22	1.01
**A-2**	**4011071**	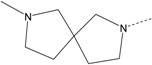	**3-CF_3_,4-Cl**	**3.59**	**3.08**
A-3	4011091	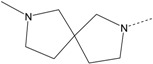	4-N(CH_3_)_2_	1.33	1.03
A-4	4021671	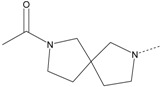	3-CF_3_,4-Cl	1.00	0.99
**B-1**	**4010301**	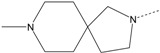	**3-CF_3_,4-F**	**2.97**	**3.08**
B-2	4010271	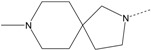	H	1.16	1.10
B-3	4010281	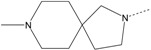	3-OCH_3_	1.20	1.08
B-4	4011531	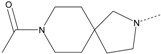	3-CF_3_	1.41	1.65
**B-5**	**4011561**	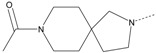	**3-CF_3_,4-F**	**2.85**	**2.66**
B-6	4011551	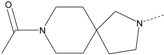	3-CF_3_,4-CH_3_	1.18	1.01
B-7	4011521	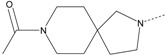	3-OCH_3_	1.18	1.02
B-8	4011571	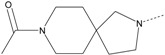	3-N(CH_3_)_2_	1.06	1.00
C-1	4010021	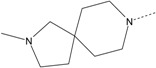	2-CF_3_	1.49	1.79
C-2	4010031	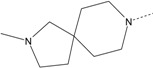	3-CF_3_	1.96	1.81
C-3	4010041	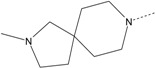	3-N(CH_3_)_2_	1.17	1.00
C-4	4011151	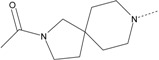	2-CF_3_	1.18	0.94
C-5	4011161	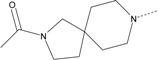	3-CF_3_	1.31	1.30
**D-1**	**4010731**	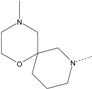	**3-CF_3_,4-Cl**	**2.37**	**2.24**
**D-2**	**4010751**	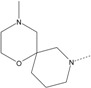	**3-CF_3_,4-CH_3_**	**2.87**	**2.74**
E-1	4010531	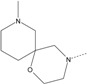	3-CF_3_	1.24	1.17
**E-2**	**4010571**	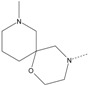	**3-CF_3_,4-Cl**	**3.34**	**2.94**

### 2.3. Structure-Activity Relationship Analysis

The substituents both in aromatic ring and spiro ring were explored by assessing several analogs containing electron withdrawing and/or electron donating groups, as well as different sizes of substituents. Our study found that electron withdrawing group trifluoromethyl substitution on the phenyl ring was essential to the Apo A-I upregulating activity. The absence of a trifluoromethyl substitution resulted in loss of the activity (e.g., A-3, B-2, B-3, B-7, B-8 and C-3). Replacement of the trifluoromethyl group with methoxy or dimethylamino substitution led to reduction of the activity among these compounds (e.g., B-4 to B-7 and B-8, C-2 to C-3). In addition, the position of the trifluoromethyl substituent affects the activity too. For example, the trifluoromethyl group on the *meta* position generally exhibited a higher potency than that of the *ortho* position (e.g., C-2 to C-1 and C-5 to C-4). Furthermore, introduction of different groups on the *para* position of the phenyl led to significant changes of activity. The addition of a *para*-chloro or *para*-fluoro on the trifluoromethyl substituted phenyl ring showed a distinct increase (2 to 3-fold) in Apo A-I upregulating activity (e.g., comparing A-2 to A-1, B-5 to B-4 and E-2 to E-1), while compound B-6 with a *para*-methyl group totally lost its activity. However, compound D-2 with a methyl group on the *para* position of the trifluoromethyl substituted phenyl ring did not decrease the activity compared to analog D-1 with chloro substitution, indicating the effect of a methyl group on the activity was unclear, and more analogs need to be investigated.

The different categories of spiro rings do not have a significant effect on Apo A-I transcriptional activity with the current data, rather for each type of analog, high activity could be attained by variation in the substitution pattern. However, substituents on the *N*-position had different effects on the activity. *N*-methyl substitution on spiro rings was more potent than its *N*-acetyl counterpart, though to differing extents (e.g., A-2 to A-4, B-1 to B-5, C-1 to C-4 and C-2 to C-5). This observation indicated that group optimization on the spiro ring might have potential to further stimulate transcriptional activity of Apo A-I. In summary, the present study illustrated that the trifluoromethyl substitution on the meta position along with a halogen substitution on the para position of the phenyl, and the *N*-methyl substitution on spiro rings, were helpful to the ability of inducing Apo A-I transcriptional activity for the substituted benzamide analogs.

## 3. Experimental

### 3.1. General

Cell culture media were purchased from Hyclone (Logan, UT, USA), fetal bovine serum (FBS) was from Gibco (Grand Island, NY, USA), dimethyl sulfoxide (DMSO) was obtained from Sigma-Aldrich (St. Louis, MO, USA), cell culture lysis reagent (CCLR) and luciferase assay substrate were from Promega (Madison, WI, USA). All other reagent-grade materials were from Life Technologies (Carlsbad, CA, USA).

### 3.2. Cell Culture

The human hepatocellular liver carcinoma cell line HepG2 were cultured in minimum essential medium with Earle’s balanced salts (MEM/EBSS) supplemented with 10% FBS, 100 U/mL penicillin, and 100 μg/mL streptomycin. The plasmid pGL4-ApoP, containing the Apo A-I promoter region from −277 to +173 bp (relative to the transcriptional start site) upstream to the luciferase reporter gene, was used to assess promoter-activation in Apo A-I gene transcription [11]. ApoP-Luc HepG2 cells, which stably transfected with pGL4-ApoP, were grown in MEM/EBSS with 10% FBS and 700 μg/mL G418. Cells were maintained in a humidified incubator at 37 °C with 5% CO_2_.

### 3.3. Compounds

The samples for screening were obtained from compound library of National Laboratory for Screening New Microbial Drugs, Institute of Medicinal Biotechnology, Chinese Academy of Medical Sciences, which consists more than 50,000 natural compounds and synthetic compounds synthesized or purchased commercially. The compound samples were dissolved in DMSO and stored in 96-well sample plates at 4 °C for assays. All compounds further evaluated in this work were purchased from WuXi AppTec (Shanghai, China). Their structures were confirmed by mass spectrometry. The purity of the compounds was analyzed by high performance phase liquid chromatography (HPLC) and all the compounds showed purity of at least 91%. Microbial Natural Products Database (MNPD, Institute of Medicinal Biotechnology & NeoSuite, Beijing, China) software was used to search for compounds of interest, those with substituted benzamides containing azaspiro rings from the compound library [17].

### 3.4. Cell-Based Screening Assay

The compound samples were evaluated in a cell-based assay for their capability of inducing Apo A-I transcription. On day 1, ApoP-Luc HepG2 cells were seeded in 96-well plates at the density of 5 × 10^4^ cells per well. The cells were incubated in MEM containing 10% FBS and 700 μg/mL G418 at 37 °C. After 6 h incubation, the culture supernatants were carefully removed and the compound samples dissolved in 0.1% DMSO at the indicated concentration and vehicle control (solvent, 0.1% DMSO) were added in the absence of FBS. On day 2, cell monolayer were washed with phosphate buffered solution (PBS) and lysed by CCLR, and then the expressed luciferase activity was measured as relative luminescence units (RLU) in Envision multilabel plate reader (PerkinElmer, Waltham, MA, USA) using luciferase assay substrate. The luciferase activities of cells treated with the test compounds were normalized to that of vehicle control to calculate the observed fold induction. In order to simplify the screening procedure, each five compounds were mixed together in equal volume proportions to give a “5 in 1” form for the primary screening. Each compound in “5 in 1” form gave a final concentration of 2 μg/mL (0.1% DMSO) in the assay. For identified “5 in 1” samples that shown more than 1.5-fold induction of luciferase activity, each compound of them (2 μg/mL) was subsequently retested with the same assay to confirm the hits, and compounds that gave inductions more than 1.5-fold higher were identified as initial hits.

## 4. Conclusions

The results in this paper revealed that some novel substituted benzamide analogs containing azaspiro rings have the ability to induce Apo A-I transcription. To the best of our knowledge, no related biological activities were reported around these analogs before. SAR exploration around analogs found changes on the molecule structures can have a significant impact on the upregulating activity. The results indicated that the trifluoromethyl substituted benzamide containing azaspiro ring is a good backbone for the further design of Apo A-I transcriptional upregulator. Taken together, this work appears to hold promise for further investigation of Apo A-I upregulators to develop more potent analogs with drug-like properties.
